# The Selection of an Appropriate Count Data Model for Modelling Health Insurance and Health Care Demand: Case of Indonesia

**DOI:** 10.3390/ijerph7010009

**Published:** 2009-12-29

**Authors:** Budi Hidayat, Subhash Pokhrel

**Affiliations:** 1 Faculty of Public Health, the University of Indonesia, Kampus FKM UI, Building F 1th Floor, Depok 16424, Indonesia; E-Mail: Budi.Hidayat@ui.edu; 2 Poverty Reduction and Economic Management, the World Bank Office Jakarta, Jakarta Stock Exchange Building Tower 2, 12th Floor, Jl. Jend. Sudirman Kav. 52-53, Jakarta 12190, Indonesia; 3 Health Economics Research Group, Brunel University, Uxbridge, Middlesex, UB8 3PH, UK; E-Mail: Subhash.Pokhrel@brunel.ac.uk

**Keywords:** health insurance, demand for health care, endogeneity, count data models

## Abstract

We apply several estimators to Indonesian household data to estimate the relationship between health insurance and the number of outpatient visits to public and private providers. Once endogeneity of insurance is taken into account, there is a 63 percent increase in the average number of public visits by the beneficiaries of mandatory insurance for civil servants. Individuals’ decisions to make first contact with private providers is affected by private insurance membership. However, insurance status does not make any difference for the number of future outpatient visits.

## 1. Introduction

Count data models have been widely used to estimate the predictors of health care demand [1–7]. Most analyses use household surveys collecting information about health care provider types and number of visits made to different types of providers during the recall period. An important issue to be considered in estimating the effects of health insurance on the demand for health care in these settings is therefore to establish whether the demand variable is generated as a discrete and mutually exclusive choice (e.g., types of providers visited in the event of an illness) or is in the form of a count or rate (e.g., number of visits made to a particular provider). The latter is usually modeled using count data models and their variants [8].

In estimating health care demand, complexities arise because the underlying behaviors driving health care utilization may have implications for the choice of the most appropriate model [7]. Further, as people demand both health insurance and health care depending on their health status, whether the model suffers from bias (due to endogeneity of the choice of insurance status and demand) should be scrutinized [9,10]. Deciding on a particular model appropriate for estimating health care demand is a difficult process that is often poorly documented in the health economics literature. The purpose of this paper is to document the complete process by which we developed guidelines for the selection of an appropriate count data model for health insurance and health care demand and to choose a particular count data model in estimating the number of outpatient visits.

In practice we will estimate the relationship between health insurance and the number of outpatient visits to public and private health care providers in Indonesia. There are previously published studies on health insurance and health care demand [9–11]; Indonesia deserves special attention as it is a developing country committed to universal coverage through a national health insurance program (NHIP). This article provides evidence on whether such a policy would be welfare-enhancing in terms of increasing access to formal health care in Indonesia.

This study also confronts directly the statistical tradeoffs associated with correcting for endogenous regressors (*i.e.*, correcting for endogeneity when it is absent results in larger standard errors, loss of precision [10], and efficiency [12]). We explore two classes of count data models. The first class is characterized by a primary equation with a discrete dependent variable. This class includes standard count data models such as restricted Poisson, negative binomial zero-inflated, and hurdle models. The second class extends the features of the first class to accommodate endogenous regressors, including instrumental variables [12,13] and generalized method of moments [14] techniques.

## 2. Health Insurance Context and Potential Source of Endogeneity

Table 1 provides summary characteristics of health insurance schemes. The Indonesian government has mandated health insurance for civil servants (*Askes*) since 1968. This scheme covers all civil servants, civil servant and armed forces pensioners, and their families and survivors. Civil servants and pensioners are automatically enrolled in this scheme. Eligible dependents include spouse and the first two children, with the cut-off age for dependent children depending on education status. The contribution is 4 percent of basic salary shared equally by employees and the government as employer. The scheme, managed by a state-owned company (*PT Askes*), covers about 14 million beneficiaries. Since *Askes* is compulsory, people may choose civil service employment based on their health status. Therefore, endogeneity of insurance status and health care demand may arise for those with less favourable health status who choose civil service employment with compulsory health care insurance benefits. Similarly, healthier workers may be more likely to choose self-employment or smaller private firms to avoid mandatory health insurance premiums.

In 1992, the government passed a Social Security Act (SSA) mandating enrolment of private employees in either privately-provided insurance schemes or the government-provided *Jamsostek* insurance scheme (which includes provident funds, death benefits, and worker’s compensation in addition to health benefits). SSA regulations stipulate that private employers with total salary costs of more than 1 million *Rupiah* per month (roughly $100 at current exchange rates) are required to enroll their employees and dependents in qualified health insurance plans managed by *PT Jamsostek*. However, the health benefit as required by the SSA is compulsory but optional, that is employers who have a better scheme than the one covered by *Jamsostek* may opt out to this scheme. This policy makes this scheme covers only 3 million workers out of about approximately 100 million workers who are eligible [15]. *Jamsostek* covers spouses and up to three dependent children less than 21 years of age with benefits include outpatient and inpatient care at both public and public health care providers. Premiums, which are capped at 3percent of basic salary for unmarried and 6percent for married employees, are paid solely by employers so *Jamsostek* is non-contributory. This policy may lead employers to choose *Jamsostek* for their low income employees with health problems while healthier employees with higher incomes will opt out. Thus, it is likely that endogeneity is an issue for *Jamsostek* membership as well.

The government also enacted the Insurance Act in 1992 which allows private insurance firms to sell health insurance products. These schemes usually offer both public and private health providers in their provider networks. The consensus estimate of the number of people with private health insurance is 5 million [15]. The health insurance literature has documented selection bias in private insurance demand; therefore one should suspect endogeneity of insurance status while estimating health care demand given private insurance [10].

Even given public policy and this menu of insurance opportunities, in 2004 only a small fraction of the Indonesian population (<15 percent) was covered by any health insurance. Motivated largely by the expectation that health insurance improves access to health care, the president signed a National Social Security Law in 2004 which will used as a basis for introducing an NHIP in the country.

## 3. Methodology

### 3.1. Model Specifications

This study estimates the relationship between insurance status and the demand for health care. The variable capturing demand is the number of outpatient visits during the four weeks prior to a household interview. The discreteness and non-negativity of this variable call for count data modeling [5–8]. Furthermore, health insurance status may be codetermined in our non-experimental dataset [10], so we utilize models that accommodate possible endogeneity of the insurance choice.

The number of outpatient visits for an individual (*M_i_*) is posited to be exponential function of health insurance (*I_i_*), exogenous variables (*x*′*_i_*), and a random error (*u*_1_*_i_*):

(1) $$M_{i}=\text{exp}(I_{i}\alpha +x_{i}^{\prime }\beta )+u_{1i}$$

where *M_i_* represents the number of visits made; *I_i_* is an individual’s health insurance attributes; *x*′*_i_* represents a vector of health, socio-economic and demographic characteristics; and *u*_1_*_i_* captures variation in an individual’s unobserved characteristics as well as random error. The exponential function ensures non-negative integers.

Maximum likelihood (ML) estimation of Equation (1) yields consistent and efficient estimations when regressors *I_i_* and *x*′*_i_* are exogenous. If health insurance status is codetermined with demand *M_i_*, outpatient visits and insurance status can be modeled with simultaneous equations [9–11]. Continuing from expression (1), an individual’s demand for insurance is modeled as follows:

(2) $$I_{i}^{*}=\gamma z_{i}+x_{i}^{\prime }\beta +u_{2i}$$

where $I_{i}^{*}$ represents an individual’s inclination to have insurance; *z* is a row vector of observable determinants that influence insurance status but not number of visits (uncorrelated with *u*_1_); *x*′*_i_* is defined as in Equation (1); and *u_2i_* is again random error also capturing unobservable insurance determinants. $I_{i}^{*}$ (an indivdual’s propensity to choose insurance) is not observed; instead we observe the categorical insurance status variable *I_i_*.

Equation (1) is the demand equation, and the reduced form Equation (2) for the suspected endogenous variable is the insurance equation. Bias in Equation (1) may arise if important unobserved determinants of insurance status *I_i_* are correlated with the random error (*u*_1_) of the demand equation. For example, those who are less healthy may have a higher than average propensity to seek insurance as well as a higher than average propensity to seek care given illness. It is likely that unobservable factors influencing demand are positively correlated with the error term *u*_1_ which would mean *u*_1_ and *u*_2_ are correlated. This would imply correlation between insurance status *I* and the error term *u*_1_. In maximum likelihood estimation, correlation between *I* and *u*_1_ (E(*u*_1_|*x*) ≠ 0) will result in a biased estimate of α in (1), the demand equation [9–11].

### 3.2. Econometrics Approaches

The dependent variable takes only non-negative integer values, and thus the family of count data models provides appropriate estimation techniques [6,8]. Poisson specifications, in which the mean of the data distribution is assumed to equal the variance, are too restrictive: it has been found in most health economics applications that the variance of the data exceeds the mean (overdispersion), the data contains a large number of zeros (see [4,5,16]), and there is a long right tail. For such data, Poisson regressions provide consistent estimates of the coefficients but not of their standard errors.

A variant of the Poisson is the negative binomial (NB) model [2]. However, with a large proportion of zeros, both Poisson and NB predictions exceed typical predictions of such models in the absence of zero values. Two common solutions accommodate excess zeros [17]. First is the zero-inflated variant of either Poisson (ZIP) or negative binomial (ZINB) distributions. The second variant is called a hurdle model, also known as a two-part model. In hurdle specifications, the first part is a binary outcome model and the second part is a truncated count data model. This study explores both ZINB and hurdle models. For the latter, we use a logit model to estimate the probability that the individual visits an OP provider (part one) and a truncated-at-zero NB model to estimate the number of visits (part two).

Following Deb and Trivedi [4], the first part of the two-part hurdle model is specified as:

(3) $$\text{P}r_{h}[y_{i}>0|x_{i}]={\left(\frac{\psi_{h,i}}{\lambda_{h,i}+\psi_{h,i}}\right)}^{\psi_{h,i}}\;\text{and}\;\text{P}r_{h}[y_{i}=0|x_{i}]=1-{\left(\frac{\psi_{h,i}}{\lambda_{h,i}+\psi_{h,i}}\right)}^{\psi_{h,i}}$$

where *y_i_* indicates the number of visits; the *λ_i_* is the conditional mean of the count, defined as *λ_i_* = exp(*x*′*_i_β*)and the precision parameter ( $\psi_{i}^{-1}$) defined as $\psi_{i}=(1/\alpha )\lambda_{i}^{k}$ with *k* as an arbitrary constant; the subscript *h* indicates parameters associated with the hurdle distribution.

The second part of the hurdle model is assumed to follow the density for a truncated negative binomial [4]:

(4) $$f(y_{i}|x_{i},y_{i}>0)=\frac{\Gamma (y_{i}+\psi_{i})}{\Gamma (\psi_{i})\Gamma ({\text{y}}_{i}+1)}{\left[{\left(\frac{\lambda_{i}+\psi_{i}}{\psi_{i}}\right)}^{\psi_{i}}-1\right]}^{-1}{\left(\frac{\lambda_{i}}{\lambda_{i}+\psi_{i}}\right)}^{y_{i}}$$

where Γ(.) is the gamma distribution function. The parameters in the second stage were estimated using the sub-sample of observations with positive values of *y_i_*, denoted as:

(5) $$\text{P}r_{h}[y_{i}>0|x_{i}]=1-{\left(\frac{\psi_{i}}{\lambda_{i}+\psi_{i}}\right)}^{\psi_{i}}$$

We use the above count data models to estimate Equation (1) with maximum likelihood techniques. In anticipation that we might have misspecified the true (but unknown) population density, we choose robust standard error procedures.

However, maximum likelihood yields consistent estimates only if regressors are exogenous. Here we suspect the regressors to be endogenous, so we consider both linear instrumental variables (IV) and generalized method of moments (GMM) estimators for both Equations (1) and (2) simultaneously. IV and GMM allow consistent parameter estimates when unobserved heterogeneity is correlated with regressors. One downside of IV is that standard errors are inconsistent in the presence of unknown heteroskedasticity, yielding invalid inference. GMM estimators using orthogonality conditions to allow for efficient estimation in the presence of heteroskedasticity of unknown form do not share this weakness. Windmeijer and Silva [14] provides a useful overview of the theoretical basis of the GMM. Mullahy [13] uses non-linear IV (or GMM) in a model of cigarette smoking behaviour.

### 3.3. Specifications Test

**Figure 1.**
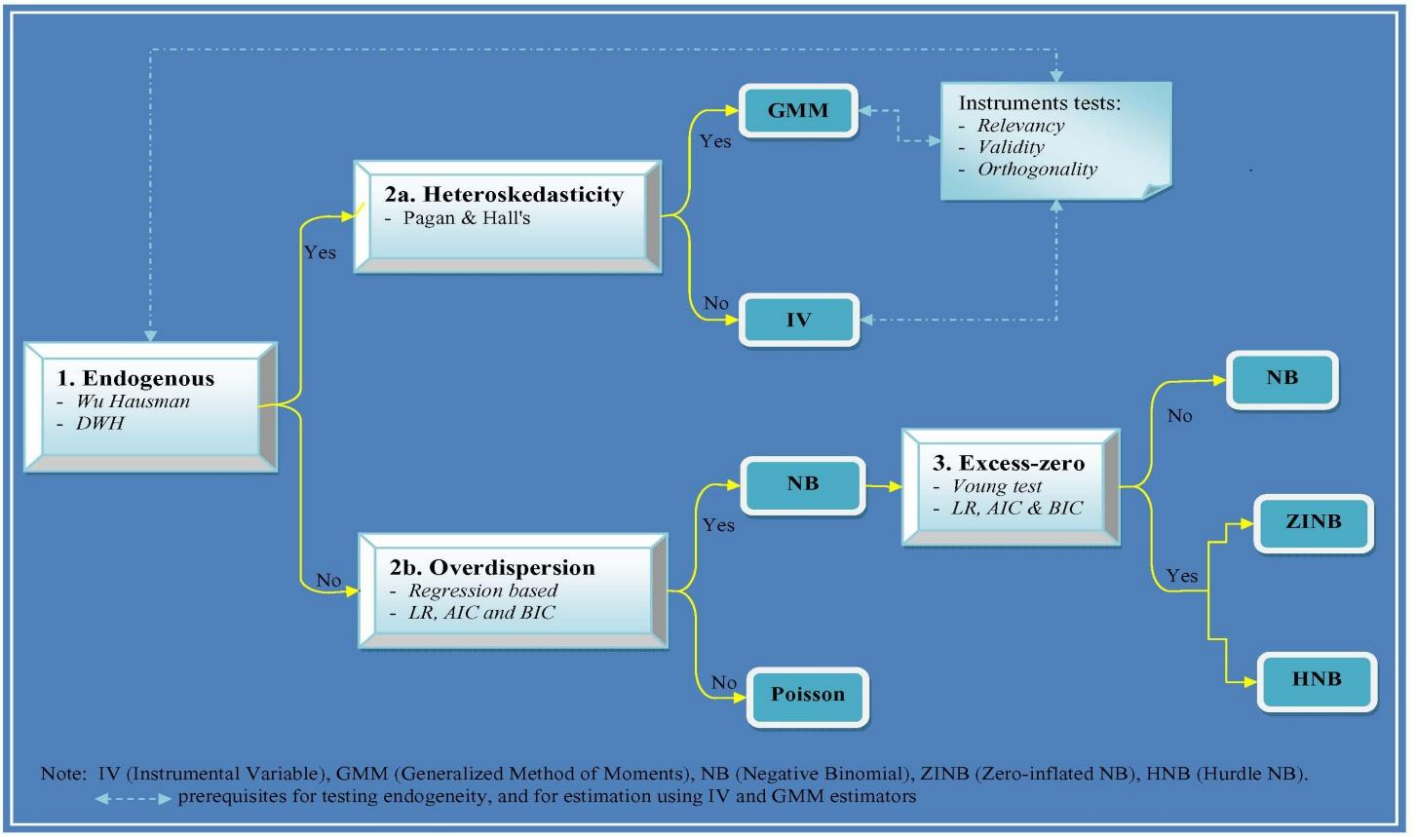
Framework to select econometric techniques for modeling the relationships between health insurance and the number of outpatient visits.

We carry out several tests in order to evaluate the overall specification of the model. Figure 1 illustrates our operational framework by summarizing what we check and what we do when assumptions are not met. It shows three main steps for choosing the most appropriate econometric technique among the six alternatives explored in this study.

The first step is testing endogeneity assumptions. To test the endogeneity of insurance status, Hausman specification tests (Wu-Hausman and Durbin-Wu-Hausman, or DWH) were carried out for each regression. In our case, this test can be interpreted as summarizing the consequences of employing different estimation methods on the same equation, not as a test for the endogeneity of regressors per se. If there is significant difference between coefficients from ML and GMM or IV, the null hypothesis of exogeneity can be rejected, suggesting either IV or GMM is necessary. Given that IV-estimated standard errors are inconsistent in the presence of unknown heteroskedasticity, we carry out various flavors of Pagan and Hall's test for heteroskedasticity [18] in step 2a. These tests are used to inform the choice between linear IV and GMM estimators; rejecting the null hypothesis of homoskedasticity suggests that GMM is preferable to IV.

Unfortunately, the consistency of the endogeneity test as well as coefficient estimates of IV and GMM depend on the validity of the instruments *z* in the insurance Equation (2). The *z* refer to the variables that have an impact, both theoretically and conceptually, on the suspected endogenous variable (insurance status) but that do not otherwise affect the demand for health care (*M*). Identification of the effect of insurance status on health care demand will be achieved if the z are uncorrelated with the structural error but correlated with the endogenous regressors, *i.e.*, health insurance variable. If the instruments are only weakly related to the endogenous variable “the resulting parameter estimates will be biased toward standard models even if the instruments are not correlated with the error term of the demand equation” [19,20].

To evaluate whether potential instruments are weak and whether the instruments are orthogonal to the error process, several tests were employed. First, the relevance of the instruments (to suspected endogenous variables) was assessed by evaluating the *R*^2^ value and the *F*-test for the joint significance of the instruments in the first-stage regressions. The first-stage regressions are reduced-form regressions of the endogenous variables on the full set of instruments and other exogenous regressors. As our models have two suspected endogenous variables, relying only on *R*^2^ and *F* statistics may not be enough to detect the relevance of the instruments. We therefore used a Shea partial *R*^2^ measure, which takes into account correlations among the instruments [21,22]. The smaller the value of the partial *R*^2^, the more inconsistent the IV estimates will be whenever the instruments are not perfectly exogenous. Even when the instruments are exogenous, a small value of the partial *R*^2^ will mean increased asymptotic standard errors and therefore reduction in the power of the *F*-test.

Second, the validity of the instruments was tested by an over-identification test [14]. Hansen's *J-*statistic and the Sargan statistic were used for GMM and IV respectively [22]. The former test is distributed as χ^2^ in the number of overidentifying restrictions. The Sargan statistic is distributed as χ^2^ with the degrees of freedom calculated as N*R^2^ from a regression of IV residuals on the full set of instruments. The joint null hypothesis of both Hansen and Sargan tests are that the excluded instruments are valid instruments (*i.e.*, uncorrelated with the error terms), and that they are correctly excluded from the estimated equation.

Finally, to satisfy an orthogonal requirement of the instruments, *i.e.*, the *z* should be exogenous, we tested a subset of instruments using the *C*-statistic [22] that allow us to test a subset of the original set for exogeneity conditions. In the case of IV, this *C*-statistic was computed as the difference between two Sargan statistics, whilst for efficient GMM, it was computed as the difference between two *J*-statistics). The *C*-test, distributed χ^2^ with degrees of freedom equal to the loss of overidentifying restrictions, has the null hypothesis that the specified variables are proper instruments.

When null hypotheses of exogenous regressors were not rejected, we used count data models that ignore endogeneity. A number of approaches were employed to select a specification that could appropriately accommodate overdispersion and excess zeros. First, to discriminate between Poisson and NB, we used a regression based approach [2] to calculate a likelihood ratio (LR) statistic as well as two traditional selection criteria based on the penalized log-likelihood, the Akaike Information Criteria (AIC) and Bayesian Information Criteria (BIC). Second, we tested the excess-zero assumption. A Vuong test was used to discriminate between the standard NB and ZINB models. The Vuong test has a standard normal distribution with large positive values favoring the ZINB model and large negative values favoring the NB model [23]. To discriminate between the HNB model and restricted NB model, following Gerdtham [24] we applied the likelihood ratio (LR) test defined as: λ = 2(ln*L*_logit_ + ln*L*_truncNB_ − ln*L*_NB_). Both AIC and BIC measures were again utilized; models yielding the smallest values of the AIC and BIC are preferred [7,16].

## 4. Data and Variables

The data for this study come from the second round of the Indonesian Family Life Survey (IFLS2) carried out by the RAND Corporation. The first round of survey (IFLS1) included interviews with 22,347 individuals from 7,224 households. The IFLS2 re-contacted the same households and succeeded in re-interviewing 93.5percent of IFLS1 households (6751 households with over 33,000 individuals). An overview of the survey is described in [25] for IFLS1 and [26] for IFLS2.

This study considers two mutually exclusive measures of OP visits: public and private providers. Not all insurance schemes offer health care services from both public and private providers and sample distributions of these variables (presented in Table 2) show that approximately 85 percent of IFLS individuals had zero visits to public OP and about 92 percent had zero private OP visits. The sample means for the number of visits to public and private OP were 0.28 and 0.15 respectively while the sample variances were 0.67 and 0.43 respectively. The ratio between the sample variance and the sample mean for the number to public and private OP visits were 2.39 and 2.87, respectively. These averages indicate the observed data is over-dispersed.

Two insurance variables, *Askes* and *Private*, enter demand Equation (1) as dummy variables. *Askes* represents mandatory insurance for civil servants and entitles beneficiaries to comprehensive health care from public providers only. *Private* represents both *Jamsostek* (insurance for private employees) and private insurance schemes and therefore may be entitling beneficiaries to care from both public and private providers. Since the effects of health insurance on health care demand might differ across income groups, an interaction term for insurance and income was included in the demand model. This interaction allows one to test whether income has different effects of insurance on the number of outpatient visits.

In demand Equation (1), the health-status vector consisted of dummy variables indicating the presence of symptoms, self-assessed general health (GHS) and severity of illness. A score assessing physical ability in the performance of daily activities (ADLs) was also included (with higher scores indicating worse ability). The vector of demographic variables consisted of age (years), gender (1/0), marital status (1/0), dummies for education, income (log natural), electricity usage (1/0), and the natural log of one-way travel time and travel cost to health facilities. To control for regional differences we used dummy variables for urban and seven regions (rural and Jakarta serving as the reference groups). Summary statistics for the variables used in the demand Equation (1) are presented in Table 3.

The endogeneity test as well as IV and GMM estimators can only be applied if one finds appropriate instruments. We propose candidate instrumental variables z that may satisfy two requirements [14]: they should be correlated with the endogenous variable(s) and they should be orthogonal to the error process. The proposed z are presented in Appendix A.

We estimated reduced form regressions of the endogenous variables on the full set of instruments (Equation (2)) using a probit model. The main objective was to generate the predicted values of insurance to be included as an additional instrument in IV and GMM techniques. The basic conclusion was that the insurance decision was more determined by income, education, age and location variables. All the proposed instruments in Appendix A except household head’s employment type had a positive correlation with choice of *Askes* insurance (*p*-value < 0.01). For *Private* insurance, only four of the proposed instruments (household head’s employment type, spouse, if active in community meetings and if housing occupied) had significant positive correlations. *R*^2^ reveals that the covariates in the *Askes* insurance estimates explained 30 percent of the overall variation, but only 20 percent of *Private* insurance variation. The joint Wald statistics shows all covariates were jointly significant in either insurance equation at the one percent level. After trying different specifications, we have selected from the proposed *z* two different subsets as final instruments for the *Askes* and *Private* equations (see Appendix A). These subsets of *z* were included in the estimation of insurance model (Equation (2)) but excluded from the demand model (Equation (1)).

## 5. Results

### 5.1. Model Selections

#### 5.1.1. Public outpatient visits

For public OP visits, the endogeneity test was rejected at 1 percent level (Table 4), suggesting that the ML estimators of standard count data model would result in inconsistent parameter estimates. Therefore, a further consideration was to select either IV or GMM estimator. The Pagan and Hall's test in GMM-estimates (χ^2^(40) = 1140.59) and in IV-estimate (χ^2^(40) = 1147.01) rejected the null hypothesis of homoskedasticity at 1 percent level. This result suggests that GMM estimator is preferable to model the number of public OP visits.

An appropriate set of instruments are prerequisites to employ the endogeneity test as well as to estimate a model using IV and GMM estimators. A number of tests were therefore employed to test the relevancy, validity and orthogonality requirements of the instruments. Table 5 provides summary statistics used in testing the relevance of the instruments. The *R*^2^ shows that the models explained a high proportion of the variation for *Askes* and *Private* insurance in both public and private OP visits. Note however that if an estimated equation yields a large value of the Partial *R*^2^ and a small value of the Shea measure, one may conclude that the instruments lack sufficient relevance to explain all the endogenous regressors and the model may be essentially unidentified [19]. In our case, the values of Partial *R*^2^ and Shea partial *R*^2^ were similar for both *Askes* and *Private* insurance, indicating that our model is well identified.

The relevance of the instruments was also investigated using the *F*-test to determine whether the instruments were correlated with the potentially endogenous variable [19]. The null hypothesis of the *F*-test that the parameters of the covariates are jointly equal to zero was rejected in both insurance types, indicating that all the instruments were jointly significant in the insurance choice equation. A conservative rule of thumb for a single endogenous regressor would suggest that a less than 10 *F*-value could be an indicator of a weak instrument [19].

The validity of the instruments was performed by applying a standard test for the over-identifying restrictions. We could not reject the null hypothesis of correct specification in public outpatient visits. The value of the Hansen's *J-*statistic (GMM-estimates) and Sargan's statistic (IV-estimates) tests was 1.921 (*p*-value = 0.383) and 1.846 (*p*-value = 0.397), respectively. This suggested that the models are reasonably well specified and the instruments are valid.

The orthogonality condition of the instruments was assessed using the *C*-statistic. The value of the test was 0.224 with *p*-value = 0.636, suggesting that the instruments used are exogenous. All empirical evidence described above led us to conclude that the selected instruments were appropriate enough to run the demand models.

#### 5.1.2. Private outpatient visits

For private OP visits, we could not reject the exogeneity hypothesis (*p*-value = 0.585). Therefore, we considered the ML estimators for standard count data models. Table 6 presents model selection criteria applied to choose the most appropriate econometric technique. We first used the results of over-dispersion measures (LR, AIC and BIC) to discriminate between the Poisson and NB models. The estimated over-dispersion parameter calculated from the NB was positive, *α* = 7.07, indicating the presence of over-dispersion. This is also corroborated by the results of the LR test −311.28 [2 × (1203.21 − 1047.57)]. As this was significant at 1 percent level, the Poisson was rejected in favor of the NB. Both AIC and BIC values also favored the NB. However, the NB ignores the existence of excess zeroes (non-users), which in private OP visits account for 92 percent (Table 2), and also treats users and non-users identically. This motivated us to consider further specifications in the regime of models that take into account excess-zeros. Possible alternatives were either ZINB or HNB.

In order to select whether NB or ZINB could be used, the Vuong test was employed. The result shows that the test was highly significant in favor of the ZINB. However, there were very large standard errors of the coefficients in the ‘inflation’ equation. This implied a definite lack of fit in case of the ZINB (results from the inflated equation are not presented here but are available from the first author).

Another option to model excess-zeros is to apply HNB. We based the comparison of this specification on the LR test and AIC values. The resulting LR test statistic χ^2^(29) for the NB model against the HNB model was 70.18 {2 × [1,047.57 − (866.94 + 145.54)]}, and was significant at 1 percent level, indicating that the HNB model could be justified well. This was also supported by the AIC, *i.e.*, this model had the smallest value of AIC among the four standard count data models. Finally, we tested the truncated Poisson model against the truncated NB model. The resulting LR statistic χ^2^(1) was 8966.5 and was significant at 1 percent level, indicating that the truncated Poisson model must be rejected against the truncated NB model. The LR test does not appear in Figure 1 since we did not explore the truncated Poisson model. The test provides an additional justification on the use of HNB.

### 5.2. Model Estimation Results

Putting together all of the above evidence, we concluded that the HNB specification is preferable to estimate the number of private OP visits. We describe below the results obtained from GMM estimation for public OP visits and HNB for private OP visits.

#### 5.2.1. Number of public outpatient visits

The first column of Table 7 presents the results from GMM estimation of the number of public OP visits. Given that the equation for the number of visits is non-linear, there is a slightly different interpretation for dummy- and continuous-variable coefficients. For coefficients on the Askes dummy variable, as *E*[*M_i_*] = exp(*I_i_α*+*x*′*_i_β*), then $\frac{E[M_{i}|I_{i}=1]}{E[M_{i}|I_{i}=0]}=\text{exp}(a)≅1+\alpha$. For α small enough, it can be interpreted as the proportionate increase in the mean of the visits owing to the *Askes* insurance effect [2]. Since *α* is 0.63, then on average individuals with *Askes* coverage would have approximately 63 percent more visits to public outpatient care. For continuous variables such as age, ADL scores, and family size, the relation that holds is [27]: $\frac{dM_{i}}{dx_{ik}}\frac{x_{ik}}{M_{i}}=\beta_{k}x_{ik}$, so the elasticity of *M_i_* with respect to *x_ik_* is linear in coefficient *β_k_*.

Coefficients on insurance dummies (*Askes* and *Private*) are positive for both schemes but significant only for *Askes* (one percent level). The coefficients on interaction terms between insurance status and income are not significantly different from zero, suggesting such interactions are not important for predicting health care demand. All health status measures have important effects on public OP demand and the effects are statistically significant (*p*-value < 0.01). Individuals who were suffering from symptoms, had higher ADL scores, and were seriously ill are more likely to have public OP visits. Relative to very good health, individuals with self-rated good and poor health are likely to have more visits. The effect of poor health is larger than the corresponding effect of good health (36 percent *vs.* 5 percent).

Women are 11 percent more likely to have more visits to public OP than men. Being married increased the average number of visits by five percent. With the exception of elementary school, the estimated effect of education is significantly negative. This indicates that higher levels of education lead to a reduction in the number of visits to public OP care (holding health status and all other covariates constant). Income elasticity for public visits was 0.03 (*p*-value < 0.01). Having electricity in the household leads to increases in public OP visits by about 11 percent (*p*-value < 0.01). The coefficients on log travel cost and time were positive but significant only in the case of the latter (*p*-value < 0.05). Urban dwellers are likely to have 11 percent fewer visits to public OP than rural residents. Jakarta’s inhabitants were likely to have a significantly lower average number of public OP visits than individuals who live in Bali, West Nusa Tenggara or Kalimantan.

#### 5.2.2. Number of private outpatient visits

The results from HNB estimation of private OP visits are presented in the second column of Table 7. The first-part estimate (binary logit) represents contact decisions while the second-part estimate (truncated-NB) represents the frequency decision. The estimated effect of the dummy for *Askes* is negative in both decision stages while for *Private* the coefficient is positive in both decision stages but only significant in the contact decision (*p*-value < 0.01). Negative coefficients on the interaction term between private insurance and income in first-part estimates imply that the probability of private OP visits, with other covariates held constant, is greater among low income groups.

With regard to health status, gender, household size, income, electricity, travel cost and time, and age, coefficients are similar to those in the public OP demand regressions described above. For example, the estimated effects of all health status measures were significantly positive in the first-part (contact decision) suggesting that individuals with a lower health status have a higher probability of visiting private OP providers.

The estimated effects of the four education dummies (elementary, junior, senior and high) were all positive and significant at 1 percent level in the contact decision. Living in an urban area increased the probability to visit a private OP by 25 percent and the frequency of private OP visits by 28 percent. East Java residents were more likely to contact private OP (36 percent) and make more subsequent visits (55 percent) compared to Jakarta inhabitants.

## 6. Discussion

This study has estimated the relationship between health insurance and the number of public and private outpatient visits in Indonesia. We have explored two econometric classes of count data models: a specification that ignores endogeneity of insurance choice and a specification that considers endogeneity of insurance choice. Although both IV and GMM estimators allow for controlling endogeneity of the insurance in the estimation [13,14], they are generally less efficient than the ML estimation of standard count data [12]. Hence, there was a trade-off between loss of precision and having biased parameter estimates [10]. Since arriving at the choice of most appropriate econometric technique is often a difficult process but not often documented in the literature in great detail, in this paper we have described criteria that helped us select most appropriate econometric technique.

We observed evidence for endogeneity (of insurance status) in the number of public OP visits. This led us to conclude that the GMM estimator is the best to model the number of public outpatient visits. Comparison of estimation results obtained from all econometric techniques explored in the study (complete results available upon request) reveals that the parameter estimates for the *Askes* insurance after controlling for endogeneity were higher than without controlling for it. This suggests that estimates of demand given insurance might depend on the empirical econometric specification used in the analysis. If the final model is not chosen based on stringent criteria as applied in this case, the calculation of premiums and prediction of financial sustainability of an insurance scheme might be underestimated. Our findings confirm empirical studies done in Ecuador and Ireland. Waters [10] found that after controlling for endogeneity of insurance, the beneficiaries of general health insurance programmes in Ecuador significantly increased their demand for curative health care by about 30 percent, whilst not controlling for endogeneity of insurance the demand effect was only 11 percent. In Ireland, Harmon and Nolan [28] found that treating insurance as exogenous, the probability of having a hospital stay was 3 percent higher for those with health insurance. When insurance was treated endogenously, the effects approximately double (6 percent).

In the case of private visits, several statistical tests suggested that the HNB hurdle specification is superior to the standard one-part specification. The use of HNB is justified by the fact that health care use in this study is measured by number of contacts instead of the total cost of all contacts [7]. Our finding is line with previous studies [3], and confirms the importance of distinguishing between factors that affect the propensity for contacting health care providers and factors that determine the volume of utilization once contact has been made [16]. Bogu [29] also suggests that count data models most commonly used are the hurdle model and the finite mixture negative binomial. The validity of hurdle specification is suspect if individuals have multiple illness episodes or multiple first contacts or the first contact belongs to an illness episode of the preceding illness [3,14,16]. Since utilization data in this study is derived from a 4-week recall period, both multiple illness episodes and multiple first contacts seem unlikely here. In addition, this study included several measures of current health conditions and an ADL score reflecting long-term health status. As they were not significant in the second stage of the HNB model, the HNB retains its superiority over other candidate models.

The HNB estimates confirm that *Askes* insurance exhibited a negative relationship in both contact and frequency decisions for private OP which is consistent with *a priori* expectations as the scheme entitles beneficiaries to services at public providers only. For *Private* insurance, the coefficients show positive relationships in both decision stages but are significant only in the contact decision. The motivation of the hurdle model comes from ‘principal-agent’ theories of the demand for health care [4]. In this regard, however, our results do not support the possibility of supplier-induced demand due to insurance. This might be the result of a strict utilization review program managed by the insurer. In addition, our results show that the main determinants of the frequency decision are need-based. This is consistent with previous studies that found no evidence of such behavior [3].

Another way to look into the evidence of supply induced demand (SID) for health care is to examine how the doctors’ density affects demand. Physician density in Indonesia is higher in urban areas. Physicians practicing in urban areas facing negative income shocks could use their dual role—both as evaluator and supplier—to induce demand [30]. In our models, the urban dummy turned out to be positive and significant in both decisions for visits to private OP, suggesting there is evidence SID where private provider competition is likely. However, future research is needed to validate our finding by including a variable that measures physician density directly.

The finding that insurance increases individuals’ propensity for health care utilization is important for policy makers, particularly in Indonesia where current debate is dominated by discussions regarding improving access to care and the introduction of national health insurance scheme. Although such findings have been reported elsewhere [9–11,28], our results are specific to two different types insurance (*Askes* with public providers only and *Private* with both public and private providers). The large effect observed in the use of private providers compared with public ones by *Private* insurance beneficiaries may be explained in the light of perceived quality of care. Theoretically, insurance reduces the effective price that beneficiaries pay for health care [30]. Insured people, given provider networks, choose the alternative that yields the highest satisfaction (utility). As this would mean increasing perceived quality and decreasing prices, the ultimate choice of provider actually reflects the relative trade-off between price and quality that individuals prefer. By offering private providers (perceived to have better quality), *Private* insurance reduces the relative price of quality, and hence the beneficiaries were more likely to use private providers. If there is a quality effect, our findings would imply that public providers may need strategies that would change people’s perceptions about their quality of care.

Another finding from this analysis bears more discussion. A negative (and statistically significant) relationship to health care demand of *Private* insurance/income interaction in the HNB estimates indicates the effects of *Private* insurance on contact decisions is more pronounced among the poor. One possible reason is that the poor have a higher price elasticity of demand, and hence the reduction in the effective price of health services due to insurance coverage increases utilization to a greater extent among poorer than among richer individuals. From a public health perspective these findings are of substantial interest, suggesting current policy on introducing the NHIP will have a stronger impact on increasing health care demand among the poor. The introduction of a demand-side subsidy to include the 76.4 million poor in the NHIP in Indonesia is supported by the findings of this study.

## Figures and Tables

**Table 1.** Characteristics of health insurance schemes in Indonesia.

**Main Characteristics**	**Health Insurance Schemes**		
	***Askes***	***Jamsostek***	***Private***
Regulation	Gov’t Regulation 69/91	Social Security Act #3/1992	Insurance Act #2/1992
Insurer	*PT Askes (Persero)*, state-owned company	*PT Jamsostek (Persero)*, state-owned company	Private insurance firms

Membership	Mandatory	Optional-mandatory	Voluntary

Eligibility	Civil servants, pensioners of civil servants and armed force	Private sector employee	Varies, depend on the contract

Beneficiaries	Spouse and 2 oldest children (<21 years if unemployed & unmarried, or <25 years if a student)	Spouse and 3 oldest children <21 years of age	Varies

Premium rate	4% payroll deduction (regardless of marital status)	Payroll deduction (single 3%; married 6%)	Varies, depend on the risk and the benefits

Premium policy	Contributory	Non-contributory	Full Contributory

Benefits, providers network	OP and IP at public providers	OP at both public and private providers; IP at public providers only	Usually OP and IP, and mostly in the private providers networks

Note: OP = outpatient health care services; IP = Inpatient health care services.

**Table 2.** Sample frequency distribution of the number of public and private outpatient visits (number of observations = 13639).

**Number of visits**	**Public outpatient visits**		**Private outpatient visits**	
	**Freq.**	**Percent**	**Freq.**	**Percent**
0	11,589	84.97	12,573	92.18
1	1,061	7.78	562	4.12
2	544	3.99	269	1.97
3	246	1.80	118	0.87
4	150	1.10	81	0.59
5	15	0.11	5	0.04
6	17	0.12	6	0.04
7	7	0.05	7	0.05
8	-		8	0.06
9	-		-	
10	10	0.07	10	0.07

*y*(mean)	0.28		0.15	
*s*^2^*y*(variance)	0.67		0.43	
*S*^2^*y/y*	2.39		2.87	

**Table 3.** Summary statistics of the variables used in the demand equation.

**Variable**	**Definition**	**Mean**	**Std. Dev.**
Askes insurance	1 if govt-employ insurance; 0 otherwise	0.098	0.298
Private insurance	1 if priv-employ insurance; 0 otherwise	0.052	0.223
Askes*income	Interaction *Askes* and income	0.165	0.775
Private*income	Interaction *Jamsostek* and income	0.073	0.419
Symptoms	1 if had ≥ 1 symptom; 0 otherwise	0.963	0.189
Score ADLs	Physical ability to perform daily activity	0.295	0.456
Very good GHS R	Very good health status		
GHS is good	General health status was good	0.788	0.409
GHS is poor	General health was bad & very bad	0.135	0.342
Serious illness	1 if had serious ill; 0 otherwise	0.127	0.333
Female	1 if female; 0 otherwise	0.574	0.495
Household size	Number of household members	5.878	2.594
Married	1 if married; 0 otherwise	0.842	0.365
No-schooling^R^	Had no education		
Elementary	Had some primary education	0.475	0.499
Junior	Had some secondary education	0.136	0.343
Senior	Had some senior education	0.196	0.397
High	Had some higher education	0.069	0.254
Age (years)	Individual age in years	36.988	11.654
Ln. Income	Log natural per-capita income (Rp)	11.099	0.855
Electricity	1 if had electricity; 0 otherwise	0.870	0.336
TravCost public	Log one way travel-costs to public health post	6.688	5.868
TravCost private	Log one way travel-costs to private health post	3.278	4.792
TravTime public	Log one way travel-time to public post	8.053	1.769
TravTime private	Log one way travel-time to private post	6.975	2.353
Urban	1 if urban; 0 otherwise	0.488	0.500
Jakarta Region^R^	Jakarta residence		
Sumatra	Lived in Sumatra	0.195	0.396
West Java	Lived in West Java	0.178	0.383
Central Java	Lived in Central Java	0.188	0.391
East Java	Lived in East Java	0.121	0.326
Bali & WNT	Lived in Bali and WNT	0.112	0.316
Kalimantan	Lived in Kalimantan	0.049	0.216
Sulawesi	Lived in Sulawesi	0.056	0.229

R is the reference group.

**Table 4.** Endogeneity tests.

**Endogeneity test:**	**Public outpatient visits**		**Private outpatient visits**	
	**Statistics**	***p-val.***	**Statistics**	***p-val.***
Hausman	F(2,13607) = 10.283	0.00003	F(2) = 0.537	0.585
Durbin Wu Hausman	χ^2^(2) = 20.584	0.00003	χ^2^(2)=1.076	0.584

**Table 5.** Tests for the relevance of instruments.

**Test statistic**	**Public outpatient visits**	
	***Askes* insurance**	***Private* insurance**
Pseudo *R*^2^		
Unadjusted *R*^2^	0.4973	0.5697
Adjusted *R*^2^	0.4962	0.5688
Partial *R*^2^	0.0561	0.0213
Shea Partial *R*^2^	0.0518	0.0197
*F*-tests:		
Wald test (a)	434.24‡	581.22‡
Wald test (b)	202.26‡	74.17‡

(a) F-test all instruments F(31,13607);

(b) F-test excluded instruments F(4,13607);

‡ significant 1%.

**Table 6.** Selection criteria of the standard count data models: private outpatient visits.

	**Poisson**	**NB**	**ZINB**	**Hurdle Negative Binomial (HNB)**	
				**1^st^ part: Logit**	**2^nd^ part: Truncated NB**
Observation (n)	13,639	13,639	13,639	13,639	1,066
LR test (29)^a^	1,203.21‡	1,047.57‡	779.22‡	866.94‡	145.54‡
−Log-*L*	5,829.72	4,735.27	4,712.28	3,271.52	1,346.48
Overdispersion test^b^	12.48‡	*n.a*	*n.a*	*n.a*	*n.a*
Vuong test c	*n.a*	*n.a*	3.3‡	*n.a*	*n.a*
Alpha^d^	*n.a*	7.07	4.17	*n.a*	0.53
AIC	11,719.45	9,532.55	9,504.56	6,603.05	2,752.95
BIC	1,970.47	588.42	533.08	661.79	
LR *vs.* Poisson test^e^	*n.a*	*n.a*	*n.a*	*n.a*	8,966.49‡

a Log ratio test of the joint significance of the regressors (in ZINB, number of regressors are 38);

b Overdispersion test for Poisson *vs.* NB model;

c Vuong test for standard NB *vs.* zero-inflated NB model;

d An ancillary parameter alpha (α) is an estimate of the degree of overdispersion in the data;

e Log ratio test for truncated NB *vs.* truncated Poisson;

*n.a* = not available, and

‡ significant at 1%.

**Table 7.** Estimation results of the GMM and HNB models.

**Variable**	**[1]**		**[2]**			
	**Public outpatient visits: GMM**		**Private outpatient visits: HNB**			
			**1^st^ part: Logit**		**2^nd^ part: NB**	
	**Coef.^a^**	**(se) b**	**Coef.^a^**	**(se) b**	**Coef.^a^**	**(se)^b^**
Askes insurance	0.631^‡^	(0.154)	−0.017	(0.135)	−0.298	(0.219)
Private insurance	0.197	(0.281)	1.274^‡^	(0.184)	0.272	(0.210)
Askes*income	0.003	(0.040)	−0.023	(0.044)	0.033	(0.064)
Private*income	−0.145	(0.114)	−0.319^‡^	(0.107)	0.075	(0.153)
Symptoms	0.287^‡^	(0.024)	3.174^‡^	(0.717)	16.314	(0.000)
Score ADLs	0.098^‡^	(0.021)	0.345^‡^	(0.079)	0.077	(0.100)
GHS very good^R^						
GHS is good	0.050^†^	(0.021)	0.414^‡^	(0.149)	0.471^‡^	(0.174)
GHS is poor	0.355^‡^	(0.032)	1.390^‡^	(0.164)	0.738^‡^	(0.216)
Serious illness	0.086^‡^	(0.024)	0.721^‡^	(0.083)	0.493^‡^	(0.170)
Female	0.118^‡^	(0.015)	0.147^†^	(0.074)	0.289^‡^	(0.086)
Household size	0.006^†^	(0.003)	0.046^‡^	(0.013)	0.029	(0.020)
Married	0.055^†^	(0.022)	−0.286^‡^	(0.102)	−0.117	(0.155)
No*−*schooling^R^						
Elementary	−0.019	(0.024)	0.362^†^	(0.142)	0.357^†^	(0.160)
Junior	−0.091^‡^	(0.033)	0.455^‡^	(0.169)	0.117	(0.186)
Senior	−0.083*	(0.046)	0.505^‡^	(0.164)	−0.246	(0.193)
High	−0.262^‡^	(0.056)	0.756^‡^	(0.185)	0.175	(0.234)
Age (years)	−0.001	(0.001)	0.003	(0.004)	−0.001	(0.004)
Ln income	0.031^‡^	(0.012)	0.383^‡^	(0.051)	−0.034	(0.062)
Electricity	0.106^‡^	(0.022)	1.003^‡^	(0.198)	0.107	(0.263)
TravCost(ln)	0.002	(0.001)	0.015^†^	(0.007)	−0.004	(0.007)
TravTime (ln)	0.009^†^	(0.004)	0.029*	(0.018)	0.065^†^	(0.026)
Urban	−0.109^‡^	(0.021)	0.228^‡^	(0.083)	0.276^†^	(0.112)
Jakarta Region^R^						
Sumatra	0.027	(0.034)	−0.327^†^	(0.127)	0.077	(0.204)
West Java	−0.047	(0.034)	−0.112	(0.116)	0.302^†^	(0.132)
Central Java	−0.034	(0.032)	0.089	(0.122)	0.141	(0.152)
East Java	0.052	(0.037)	0.509^‡^	(0.135)	0.554^‡^	(0.157)
Bali & WNT	0.076^†^	(0.036)	0.150	(0.143)	0.015	(0.170)
Kalimantan	0.136^†^	(0.054)	−1.080^‡^	(0.257)	0.360	(0.425)
Sulawesi	0.042	(0.042)	−0.648^‡^	(0.242)	0.092	(0.289)
Constant	−0.730^‡^	(0.129)	−12.743^‡^	(1.002)	−17.923^‡^	(1.097)

Number observations	13639		13639		1066	

a The estimated parameters; superscript ^‡^,^†^, and ^*^ indicate significance at 1%, 5%, and 10% level, respectively;

b Robust standard errors in (parentheses);

R is the reference group.

## Appendix A.

**Table:** The proposed instruments (z*_i_*) and the selected z*_i_*.

**The proposed instruments z*_i_***	**The selected z*_i_* in estimating the number of outpatient visits to:**	
	**Public providers**	**Private providers**
If household head government employee (1/0)		√
If household head private employee (1/0)	√	√
If active in community meetings (1/0)		
If active in cooperative meetings (1/0)		√
If active in women group organizations (1/0)		√
If housing occupied (1/0)		√
If spouse (1/0)	√	√
The predicted value of *Askes* insurance*	√	√
The predicted value of *Private* insurance*	√	√

* generated from the prediction of the first-stage regression estimation (Equation 2); marked √ indicates the proposed z*_i_* was selected in the IV and GMM estimations.
